# OpenWeedLocator (OWL): an open-source, low-cost device for fallow weed detection

**DOI:** 10.1038/s41598-021-03858-9

**Published:** 2022-01-07

**Authors:** Guy Coleman, William Salter, Michael Walsh

**Affiliations:** School of Life and Environmental Sciences, Sydney Institute of Agriculture, The University of Sydney, Brownlow Hill, NSW Australia

**Keywords:** Plant sciences, Engineering

## Abstract

The use of a fallow phase is an important tool for maximizing crop yield potential in moisture limited agricultural environments, with a focus on removing weeds to optimize fallow efficiency. Repeated whole field herbicide treatments to control low-density weed populations is expensive and wasteful. Site-specific herbicide applications to low-density fallow weed populations is currently facilitated by proprietary, sensor-based spray booms. The use of image analysis for fallow weed detection is an opportunity to develop a system with potential for in-crop weed recognition. Here we present OpenWeedLocator (OWL), an open-source, low-cost and image-based device for fallow weed detection that improves accessibility to this technology for the weed control community. A comprehensive GitHub repository was developed, promoting community engagement with site-specific weed control methods. Validation of OWL as a low-cost tool was achieved using four, existing colour-based algorithms over seven fallow fields in New South Wales, Australia. The four algorithms were similarly effective in detecting weeds with average precision of 79% and recall of 52%. In individual transects up to 92% precision and 74% recall indicate the performance potential of OWL in fallow fields. OWL represents an opportunity to redefine the approach to weed detection by enabling community-driven technology development in agriculture.

## Introduction

In Australian large-scale conservation cropping systems, where growing season rainfall generally limits crop yields, fallow phases are incorporated in rotations to conserve soil moisture for subsequent crops^1,2^. These phases provide an opportunity for improved weed and disease control^3,4^ and nutrient conservation^5,6^. To maximise stored soil moisture growers prioritise maintaining weed free fallows^7^, which frequently results in repeated applications of whole-field herbicide treatments to low density (< 1.0 plant 10 m^−2^) weed populations. The development of reflectance and fluorescence-based weed detection technologies that enabled site-specific weed control (SSWC) of low weed densities in fallow fields, began in the early 1980s^8–11^ (for an overview see review by Peteinatos et al.^12^). As all living plants in fallows are considered weeds, these detection systems use spectral filters and photodiode sensors to detect chlorophyll fluorescence^10^. For over two decades sensor-based weed detection has been used in the development of spot-spraying systems that are now widely used for fallow weed control by Australian growers^13,14^. These spot-spraying systems can effectively control low density weed populations to realise weed control savings of up to 90%^15^.

The effective application of site-specific treatments has enabled a more efficient approach to fallow weed control and created interest in the development of this approach for in-crop use. In addressing the threat of herbicide resistant weed populations in their production systems, Australian growers have been reducing in-crop weed densities through the diligent use of diverse weed control treatments^16^. Low in-crop weed densities have increased the interest in specifically targeting these weeds to achieve similar savings in weed control inputs as those realised with fallow SSWC. However, sensor-based fallow weed detection technologies are only suitable for detecting growing plants, with little opportunity for further development to discriminate between crop and weed plants^12,13^. The use of digital, visual spectrum imagery has long been identified as an approach to collect the type and quantity of data required for accurate discrimination between crop and weed plants^17,18^. Imaging sensors offer detailed data streams with standard, visible spectrum digital cameras providing three channels (red, green and blue [RGB] images) of spatial and spectral intensity information. The richer data collected by these systems can be used for the more challenging task of in-crop weed recognition, with substantial research efforts focussed on developing this opportunity for large-scale systems^19^.

The introduction of durable, small-scale, low-cost computing and digital camera systems has created the potential to develop simple algorithm-based weed detection systems for fallow weed control in large-scale cropping systems. The Raspberry Pi is an example of a low-cost single board computer that was developed as a teaching resource to promote computer science in schools^20^. When coupled with a digital camera, the Raspberry Pi can be used in simple computer vision related tasks, including fallow and in-crop weed detection. For fallow weed detection, where differences between the target weed and soil or stubble background are clear, simple plant colour-based detection methods may be sufficient for weed detection. A number of studies have developed weed detection algorithms based on specific plant features such as colour^18,21,22^, shape^23,24^, texture^25,26^ or a combination of these features^27–29^. Importantly, non-machine learning algorithms typically have lower computational requirements and perform faster on less powerful processors, such as the Raspberry Pi, improving the likelihood of real-time operation in large-scale cropping systems^30,31^. Critically, the computational requirements of the algorithm determine the framerate and hence the real-time capability of the device. Coupling low-cost hardware with an open-source and community led approach to software development provides an opportunity to rapidly progress the development of these technologies for weed detection in cropping systems. Similar approaches have been effective in industries including medical research^32^, autonomous vehicles^33^, machine learning^34^ and implemented by software companies such as Microsoft^35^.

Image-based weed recognition for SSWC represents a fundamental shift in the approach to weed control for large-scale growers in Australia. A practical understanding of the limitations and realistic opportunities of this new approach by growers and the wider weed control community is an important aspect of the effective development and use of this new technology. Growers are generally regarded as Bayesian learners, where an understanding of technology is best achieved through practical “hands-on” use^36^. The general objective of this research was to develop and validate an open-source and low-cost option for weed detection in fallow fields, with potential for upgrades and improvement with future software and hardware innovations. Specifically, we aimed to (1) develop the OpenWeedLocator (OWL) as a low-cost image-based weed detection system; (2) advance weed control industry understanding and familiarity in the use of digital image-based weed detection systems; and (3) validate the baseline efficacy of OWL using colour-based algorithms for fallow weed detection.

In the following section, OWL configuration and the parameters under which the design process was guided are described. The validation of the OWL for fallow weed detection using colour-based algorithms and the implications of the device for SSWC are discussed in further sections. All software, hardware designs and a guide to build the OWL are available at https://github.com/geezacoleman/OpenWeedLocator.

## OWL configuration and system development

### Establishing design parameters and configuring OWL for weed detection

Five key design parameters for the OWL units were identified that facilitated the development of OWL units within the scope of improving community understanding and familiarity with use of imaging and algorithm-based weed detection systems. Specifically, the OWL platform needed to include (1) low-cost and accessible “off-the-shelf” hardware components; (2) simple designs with minimal use of specialised electrical tools; (3) 3D printable enclosures and mounts for accessible and customisable production; (4) modular and simple image-based software for ease of contribution and explanation; and (5) validated performance using simple and existing colour-based algorithms in fallow scenarios at relevant levels for weed detection, addressed in the algorithm assessment component of this research.

**Figure 1:**
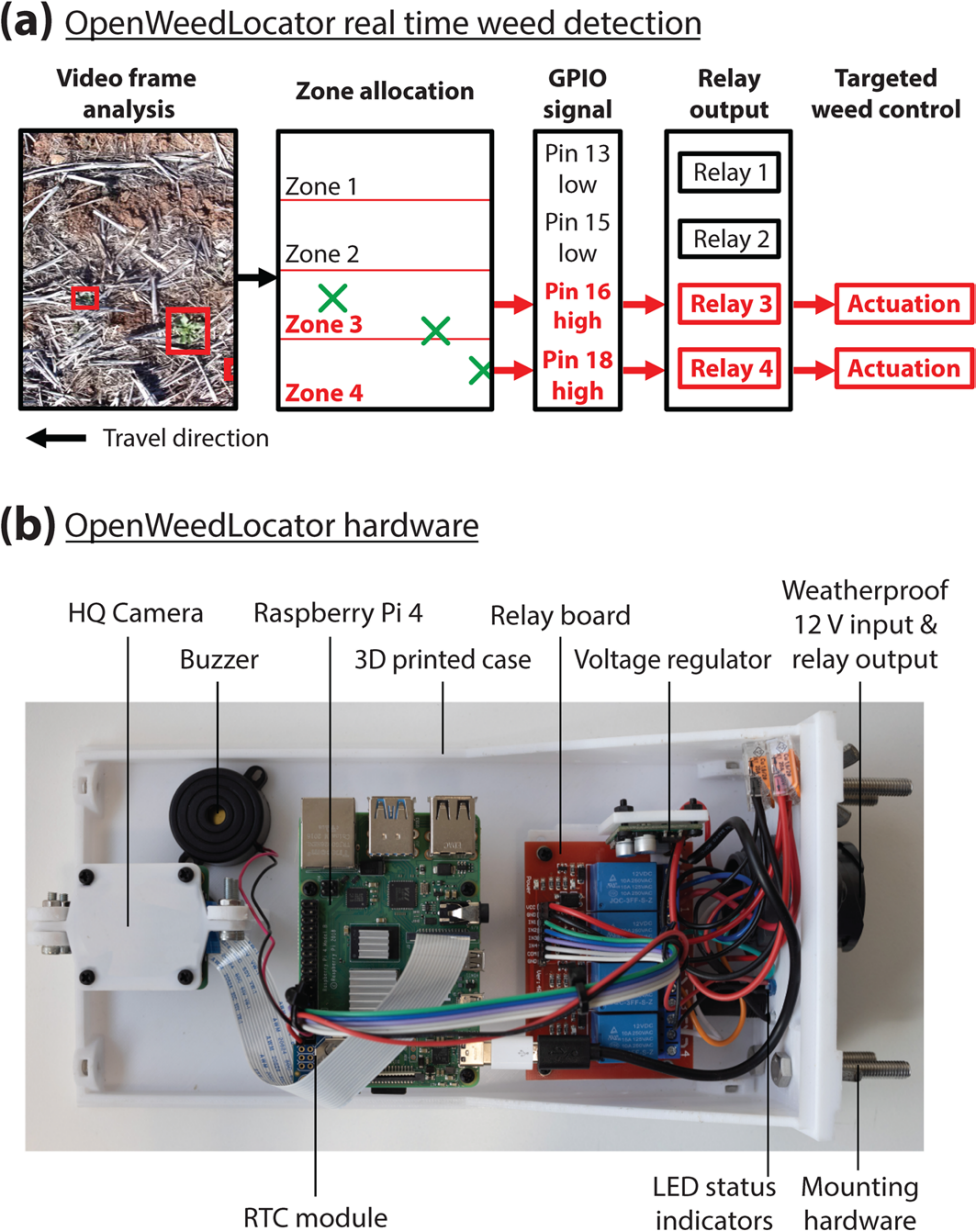
Overview of the OpenWeedLocator (OWL) (**a**) software and (**b**) hardware, which combines weed detection with an actionable output. Detection is achieved with a Raspberry Pi 4 8 GB and HQ camera with actuation achieved using 12 V relays on the relay control board. A real time clock (RTC) module is used for accurate timekeeping. A 12 V DC source is required to power the system, with a voltage regulator providing 5 V power for the Raspberry Pi computer. A six-pin weatherproof connector is used to connect the OWL unit to the 12 V power supply and to connect the relays to four external devices. The buzzer and LEDs provide status information.

The image processing components of OWL consist of a Raspberry Pi 4 (Raspberry Pi Foundation, Cambridge, UK) 8 GB computer coupled to a Raspberry Pi HQ Camera with a Sony IMX477 CMOS sensor. This camera provides a maximum sensor resolution of 4056 × 3040 pixels with a 7.9 mm optical format and a rolling shutter. Images are resized to 416 × 320 pixels to ensure high processing throughput on the Raspberry Pi platform. The HQ camera connects to the Raspberry Pi using the camera serial interface (CSI) cable and port, whilst operating the camera is completed with the inbuilt picamera Python API. A 6 mm focal length C/CS lens (Raspberry Pi Foundation) was used with the camera, providing a 1 m horizontal field of view (FOV) on the ground at an operational height of 0.82 m above the soil surface. Camera settings, including white balance, exposure and shutter speed, remained automatic as default, whilst focus was set manually during setup of the system. Based on the detection outputs from the selected algorithm, the pixel coordinates of each detection determine the allocation to one of four 25 cm wide zones covering the 1 m on-ground FOV. A unique general purpose input/output (GPIO) pin is assigned to each zone, which is activated for a specified duration if the weed is detected within that zone. A generic and low-cost relay control board enables the low current, low voltage GPIO signal to drive higher power devices including, but not limited to, water and hydraulic solenoids for targeted weed control, such as spot spraying or site-specific tillage (Fig. 1). The system is powered by a 12 V DC input with a voltage regulator (POLOLU-4091; Pololu Corporation, Las Vegas, NV, USA) providing 5 V power to the Raspberry Pi and associated components. Although optional for the purposes of weed control, we included a real time clock (RTC) module (ADA3386; Adafruit Industries, New York, NY, USA), a buzzer and several LEDs in our test system for timekeeping, system alerts and monitoring system status, respectively. The Python code and detailed installation instructions are provided in the OWL open-source repository (https://github.com/geezacoleman/OpenWeedLocator).

### System development and an open-source platform for community engagement

OWL enables weed detection and potential targeting by integrating the within-image location of each detected weed with a defined channel (GPIO pin) on the Raspberry Pi and subsequently a relay on the connected relay board. To address the first three design parameters, four OWL units were assembled using simple, “off-the-shelf” and low-cost items, with an approximate cost of AU$400 per unit. The critical components in the system, namely the Raspberry Pi, camera, voltage regulator and relay control board are easily accessible due to their extensive use in other industries. Activating an OWL unit requires connection to a 12 V DC power source, a voltage commonly available on most vehicles and powered farm equipment. The design is simple to assemble with minimal soldering or specialist tools required. It is also modular, with the ability to replace individual components and maintain functionality without changes to form or software. For example, given the use of generic GPIO-based trigger of the relay control board, the Raspberry Pi could be swapped with other embedded computers such as the Jetson Nano or Jetson Xavier for access to more powerful processing without substantial changes to software or hardware. Internally, all electrical connections are made using terminal blocks and press fit connections. The OWL enclosure and all mounting parts are 3D printable and all 3D model files are freely available for use and customisation (https://www.tinkercad.com/things/3An6a3MtL9C). An important aspect of using widely accessible components is the existing widespread support by the ‘maker’ community with respect to more general issues and upgrades of Raspberry Pi hardware and Python software. These resources improve problem solving availability for end users with reduced risk of obsolescence.

The fourth design parameter, the modularity, customisability and interpretability of the OWL detection and actuation software, was the focus of software development. The Python language-based software enables the selection of all tested algorithms (see “Methods”), adjustment of threshold parameters, minimum detection size requirements (in pixels) and other features, such as frame saving for dataset development, video recording and visualisation of the detection process. The incorporation of new algorithms is fundamental to ongoing development. The modular design of the software allows new algorithms to only require an image as an input and return a grayscale image as an output. This allows further improvements or additions of new algorithms to be made easily without restructuring of the code base.

The OWL system is supported by an open-source software repository to create a pathway for feedback and ongoing development, whilst providing a practical device on which to learn about image-based weed detection. Specific instructions and extensive guides are available for self-guided assembly, with the widely used platform GitHub selected as an avenue to engage with community feedback and development whilst providing accessibility to the code and instructions (https://github.com/geezacoleman/OpenWeedLocator). The online platform also supports logging of issues and tracking of changes over time with the release of new software versions as improvements are incorporated.

## Validation of OWL with colour-based weed detection algorithms

**Figure 2:**
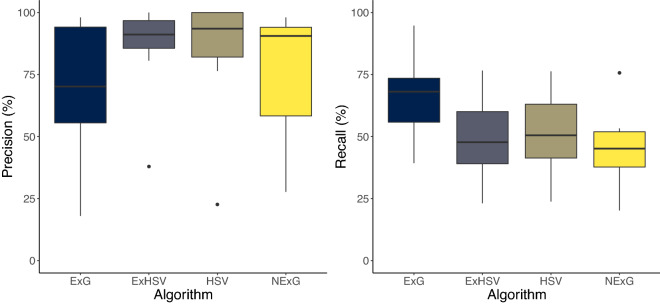
Comparison of weed detection performance metrics precision and recall across ExG, NExG, HSV and ExHSV algorithms. Values presented are based on all seven field sites visited to indicate variability. Boxplots present the median and interquartile range with the boxes, and the range and outlier points (if more than 1.5 times the interquartile range) with the lines and points.

The four algorithms used to validate OWL, namely excess green (ExG), normalized ExG (NExG), hue saturation value (HSV), and a combined ExG and HSV (ExHSV), performed equally well across the seven fields on the low-cost, Raspberry Pi-based OWL hardware, with no statistical differences found for either the precision or the recall (P > 0.05) (Fig. 2). The mean recall—the percentage of true weeds detected—was 52.2 ± 5.1% (mean ± SEM) whilst the mean precision—the proportion of detections that were correct—was 78.8 ± 3.6% (mean ± SEM). Across the seven transects, median recall rates for ExG, NExG, HSV and ExHSV were 68.1, 45.5, 47.0 and 47.7%, respectively. Median precision values were 70.2, 90.6, 96.6 and 91.1%, respectively. Although no algorithm clearly outperformed the others, ExG appeared more sensitive to weed detection, albeit with reduced precision. HSV and ExHSV appeared to have lower rates of false positive detections (Fig. 2). In five of the seven transects, the maximum recall was observed with the ExG algorithm (Table 1). In all five daylight transects, ExHSV had the maximum precision, whilst HSV had precision of 100% in both night-time, artificially illuminated transects. The normalised ExG algorithm (NExG) did not outperform the other three algorithms in any of the transects for any performance metric, with complete loss of detection under NIGHT2 conditions using the parameters tested.

**Table 1:** Summary of algorithm performance across seven field test sites during the day (n = 5) and night (n = 2) with artificial lighting using precision and recall.

Algorithm	ExG		NExG		HSV		ExHSV	
*Location*	Precision (%)	Recall (%)	Precision (%)	Recall (%)	Precision (%)	Recall (%)	Precision (%)	Recall (%)
*HEN1*	18.0	**94.7**	27.7	75.8	22.6	76.3	**37.9**	71.6
*HEN2*	46.5	**73.2**	90.2	53.3	87.7	50.5	**94.4**	54.4
*WAG1*	91.8	**73.8**	95.1	47.8	96.6	39.9	**99.1**	47.7
*WAG2*	70.2	**68.1**	90.9	36.1	91.0	48.8	**91.1**	43.4
*COB1*	98.0	39.3	98.0	20.2	**100**	**47.0**	**100**	23.1
*NIGHT1*	64.5	**48.9**	47.7	42.5	**100**	23.8	80.6	34.7
*NIGHT2*	96.4	62.7	–	–	**100**	35.7	90.6	**76.6**

The highest result for each performance metric within each field is bolded.

Across the seven fields, the performance of the weed detection algorithms varied substantially (Table 1). The minimum precision for all algorithms tested was found in field HEN1 whilst the maximum precision was found in WAG1. HEN1 had substantial canola stubble present, which under strong sunlight conditions resulted in bright white reflections and frequent false positive detections (indicated by mean precision of 26.6 ± 4.3%). WAG1 on the other hand had a high weed density, sparser lupin stubble and red soil, resulting in a reduced rate of false positive detections (indicated by mean precision of 95.65 ± 1.5%).

**Figure 3:**
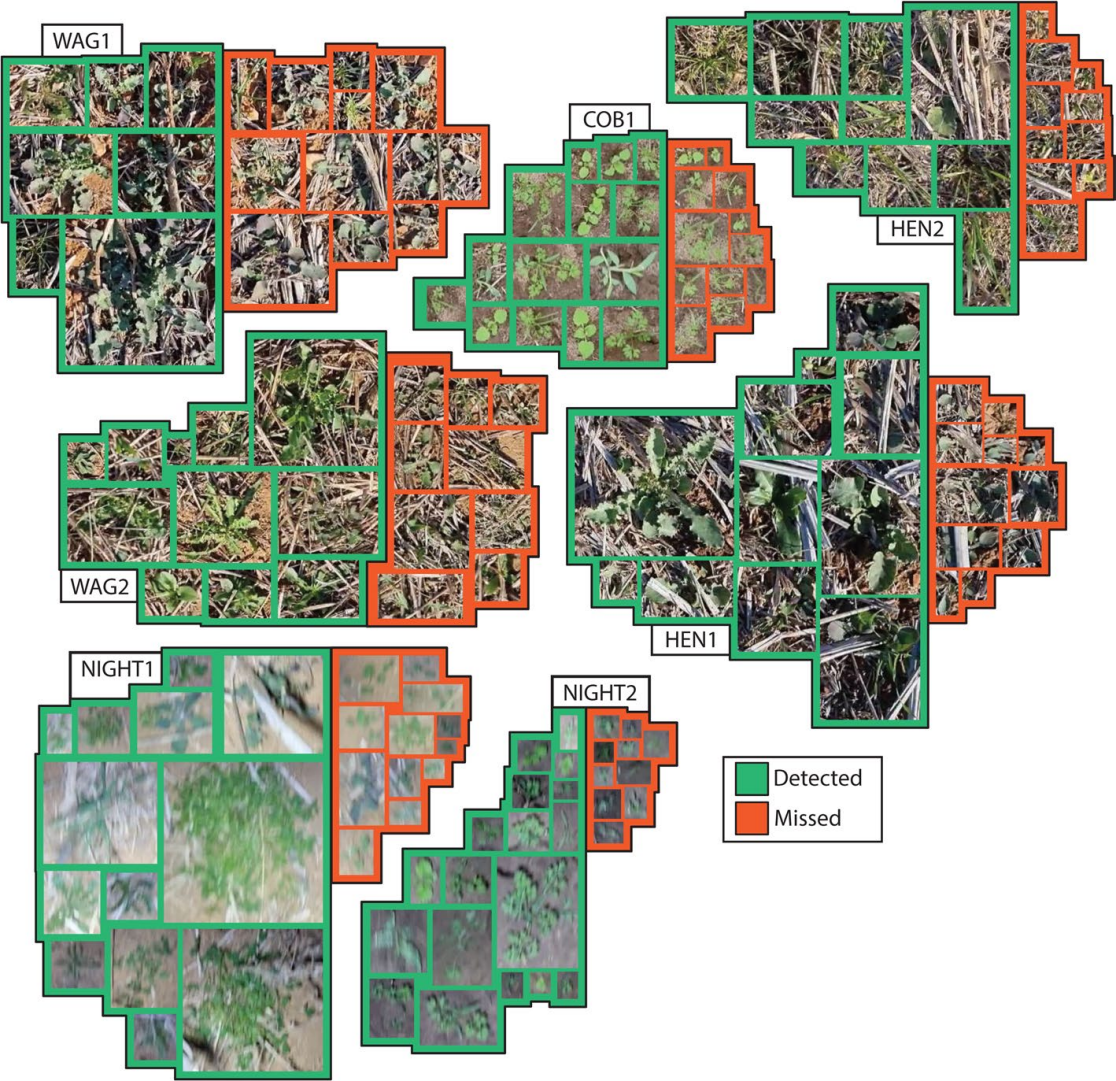
Representative weeds that were either correctly detected (green) or missed (red) at each of the seven field sites based on the ExHSV algorithm. Images of weeds shown were taken directly from concurrent video collected with a Samsung S8 phone camera and have not been rescaled, suggesting relative size is accurate.

Given the colour-based nature of the algorithms, small annual sowthistle (*Sonchus oleraceus*) that was grey-green or purple-green in colour was not well detected (Fig. 3). Similarly, the thin leaves of small rigid ryegrass (*Lolium rigidum*) and other grass weeds, including volunteer wheat (*Triticum aestivum*) and barley (*Hordeum vulgare*), were poorly detected and often missed. Small, medium and large broadleaf weeds with strong green colouring, including wild radish (*Raphanus raphanistrum*), sowthistle, volunteer canola (*Brassica napus*), volunteer faba bean (*Vicia faba*), billygoat weed (*Ageratum conyzoides*) and catsear (*Hypochaeris radiata*), were confidently detected, however, obstruction by heavy stubble increased the likelihood of the weed being missed.

Frame rates were recorded to assess the processing demand of each algorithm and the likelihood of real-time operation. This is important for OWL, given the relatively limited processing power of the Raspberry Pi. HSV had the highest framerate of 35.3 FPS (P < 0.01), indicating that it had the lowest processing requirements. ExG had the second highest framerate of 22.6 FPS (P < 0.01), whilst ExHSV and NExG were the slowest of the algorithms with framerates of 15.4 FPS and 16.6 FPS, respectively.

## Discussion

OWL capitalises on recent developments of low cost and small form factor computing systems, digital imaging sensors, so-called “maker” hardware and open-source software packages. The original Raspberry Pi computer was released in 2012 with the explicit focus of teaching basic computer science to ‘young people’ and igniting interest in programming^20^. Technological advancements to the Raspberry Pi system in the years since, including more powerful processors, increased memory, and networking capabilities, have allowed for increasingly complex projects to be developed using the system. This has led to a large online community of “makers”, who have found a multitude of uses for the Raspberry Pi, ranging from environmental monitoring^37^ to cloud computing infrastructure^38^, and who, with the help of platforms such as GitHub and StackOverflow, can provide support for hardware and software related issues and improve the development process^39^. The Raspberry Pi was an obvious choice for preliminary development of OWL, due to its widespread availability, low-cost, educational roots, interfacing options (through GPIO pins) and computational power, as well as existing and extensive online support communities. Coupling the GPIO pins of the Raspberry Pi with a relay control board makes identifiable interactions between the camera input, image-based detection output and actuation. The use of the OWL for SSWC requires coupling with external actuators such as solenoids for spot spraying or targeted tillage^40^. The OWL platform combines simple assembly and software designs with practical fallow field detection outcomes.

OWL represents a novel opportunity for community-driven development of weed recognition capability using existing ‘off-the-shelf’ hardware and simple yet effective image-based algorithms. The combination of the OWL device, supporting documentation and repository create a channel for practical education of key image-based weed detection and actuation concepts for growers and the wider weed control community. The topic is of particular importance now, given the emergence of image-based in-crop weed recognition technologies. OWL has been designed as a community focussed educational platform that will grow over time with initial baseline validation performed in the present research. The platform relies heavily on the principles of first- and second-order learning^41^. The term “first-order learning" refers to the education of growers, where users of a new technology learn with hands-on experience, in this case how image-based weed detection systems work by building and using an OWL unit. Early exposure to novel precision agricultural technologies in this manner has been shown to be strongly correlated with the adoption of new precision agricultural tools^36^. The term “second-order learning” refers to the education of SSWC technology developers based upon feedback from users and specific user needs. This has also been coined the “learning by using” approach^42^. The open-source availability facilitates such an approach, allowing continual development of OWL, weed recognition technologies more generally, and ongoing educational opportunities for the broader weed control community. Similar open-source approaches to software and hardware development have been used successfully in other industries, including machine learning^34,43,44^, medical sciences^32,45^, scientific imaging^46,47^ and autonomous driving^48,49^ to improve development speed, adoption of technology, reproducibility and error management^35,50^. In agriculture, practical implementations of grower-ready open-source systems are more limited, though include AgOpenGPS^51^ and FarmOS^52^, and now OWL. The GitHub platform selected for engagement with growers enables both the dissemination of instructions in an easy-to-read format and facilitates community contribution through licensed replication, adjustment and change tracking and has been successfully used in the transition of closed source to open source software^35^. The approach is in line with similar open-source dataset and code publications such DeepWeeds^53^, pybonirob^54^ and OpenSourceOV^55^.

The algorithms chosen to validate the baseline in field performance of OWL represent widely used methods of colour segmentation^56^ or vegetation index generation^18^. Despite the challenging scenarios and low cost of hardware, maximum rates of precision of 100% and recall of 94.7% were recorded by ExHSV/HSV and ExG algorithms, respectively, demonstrating clear potential for the use of image-based weed detection in fallow systems. Precision and recall means across all fields were lower than expected, likely a result of established limitations of using colour-only algorithms in highly complex and diverse environmental conditions with variable weed colour^19,57^. Based on qualitative assessment of weed characteristics, it is likely that variable weed appearance, in particular stressed, purple-green annual sowthistle and thin grass weeds on diverse soil and stubble backgrounds, contributed to the reduced performance of colour-only algorithms. Factors such as image blur, resulting from slow shutter speeds and the rolling shutter of the Raspberry Pi HQ camera are likely to have contributed to low recall. High image blur results in the green pixels from small or thin-leaved weeds being averaged with neighbouring background pixels, resulting in missed detections and poorer performance of colour-based algorithms^58^. Immediate improvements would likely be observed with more advanced global shutter cameras and brighter, more uniform illumination for faster shutter speeds^59^. Deep learning algorithms have been found to be more tolerant to blur, lighting and colour variability^60^, though would likely run too slow on Raspberry Pi devices and require large image datasets for training and generalization. Exploiting the colour differences between growing weeds and background is a simple method to validate the OWL system. Whilst substantial advances in useability of deep learning systems continue to be made, supporting these algorithms increases both the complexity and cost of the system, with a requirement for more powerful embedded computers. Nonetheless, the modular nature of the OWL system allows future versions to utilise more powerful processors running more advanced algorithms.

Whilst no differences (P > 0.05) were found among algorithms when compared across all seven transects, trends in performance and algorithm variability suggested field-scale differences in performance would be likely. ExG appeared more sensitive to weed detection than the other algorithms, which would result in fewer weeds being missed. The low variability in precision of ExHSV, coupled with the ability to refine sensitivity in two colour spaces suggests it is a better option for large-scale weed detection in environments where weeds are large and green. Similarly, Kawamura et al. found combining HSV and ExG features improved performance of a machine learning model over other models trained on HSV alone^61^. Using ExG instead of NExG in the composite ExHSV algorithm may be advantageous based on the results presented here, however, others have found non-normalized RGB chromatic coordinates to be highly variable^18^, resulting in poorer performance. Additionally, managing bright reflection from white stubble is critical in environments with bright sunlight, where the use of specular reflection management approaches such as that developed by Morgand and Tamaazousti^62^, would likely reduce false positives in heavy stubble conditions. Nevertheless, under current settings it appears that the precision of ExHSV offers a reduced risk of excessive false positives in stubble. Further analyses with more field trials in defined environments would improve our ability to confidently determine the most effective algorithm, however, the adjustment of colour-based algorithms to suit individual environmental circumstances is a well-known drawback of these approaches^57^. This may be required for consistently effective use as a fallow weed control tool. The benefit of OWL is that there are opportunities to include additional reduced sensitivity options when used under brightly sunlit, heavy stubble conditions, such as those at field site HEN1, where precision across all algorithms is substantially reduced. Whilst the very high precision recorded for all algorithms at the WAG1 field site is encouraging, the result is likely due to the high density of weeds, where the frame is already filled by green objects with few opportunities for false positives. Based on the experiences of other industries, the use of image-based weed detection is likely to expand as a result of community engagement from open-source availability, leading to rapid progression in SSWC for fallow crop production systems. Importantly, the detection of green plants does not limit the OWL to fallow spraying. Inter-row use of the device for weed control in wide row crops or the site-specific application of weed control, fertiliser, desiccants and irrigation in crops may also be viable uses, demonstrating the wide-ranging potential of the device.

On embedded devices such as the Raspberry Pi, the processing speed, as measured here as the framerate of each algorithm, is an important metric to determine maximum possible forward speed for real-time use. The low framerates at which ExHSV and NExG run highlight the increased computational demand of these algorithms compared to HSV and ExG. HSV ran at the highest framerate, which is likely due to the binary output image (black and white only) not requiring an additional computationally expensive adaptive threshold. This finding is contrary to Woebbecke et al.^18^, where HSV was found to be more computationally expensive, however, the evaluation in that study was based purely on defined thresholds rather than a combination of both defined and adaptive thresholds. Previous methods of green-based differentiation have employed Otsu’s thresholding^29^, where the appropriate threshold value is determined algorithmically based on image content. In large-scale, fallow scenarios where weeds are infrequent, relying solely on adaptive thresholds such as this may result in false positives when no clear green signal is provided by the selected algorithm. The combination of defined and adaptive thresholds in ExG, NExG and ExHSV was used in this study to better determine weed presence where weeds are infrequent. It is highly unlikely that framerate is a limiting factor for OWL, with real-time operation in large-scale systems (forward speed dependent) observed at framerates above 6 FPS for other systems^29–31,53,63^, with current commercial systems operating between 16 and 17 FPS for forward speeds between 2.67 and 6.67 m s^−1^^64,65^. The performance of algorithms in the field is likely also dependent on the ambient lighting conditions, which in turn influence the blurriness of the video feed. Optimising these factors and measuring impacts will be important in determining the most effective weed detection algorithm for fallow scenarios.

## Conclusion

The development of lower cost, smaller form factor and higher power computing is generating opportunities to deploy more accessible weed recognition technologies and embrace the potential for education and engagement that open-source software and hardware brings. The validation of OWL presented here has immediate applications as a low-cost image-based fallow weed detection device for large-scale crop production systems. The open-source and community-driven nature of the system enables ongoing development and opportunities to further increase the complexity of detection possibilities and reliability by upgrading the algorithms, embedded computer and camera hardware, and system settings. The results presented here of colour-based algorithms in seven separate field transects under both day (full sun, overcast) and night (artificial lighting) demonstrate the baseline potential for the OWL unit, with individual field performance at levels equivalent to other fallow detection systems. The high precision of the ExHSV combination algorithm suggests it may be relevant for use with large weeds in stubble, where the green signal is strong and false positives are undesirable. In contrast, the higher recall of the ExG algorithm suggests it may be better applied detecting smaller weeds and reducing misses. OWL sets an open-source path for the weed control industry, to assist in the affordable, site-specific and effective control of weeds in a variety of scenarios.

## Methods

### Field data collection

**Figure 4:**
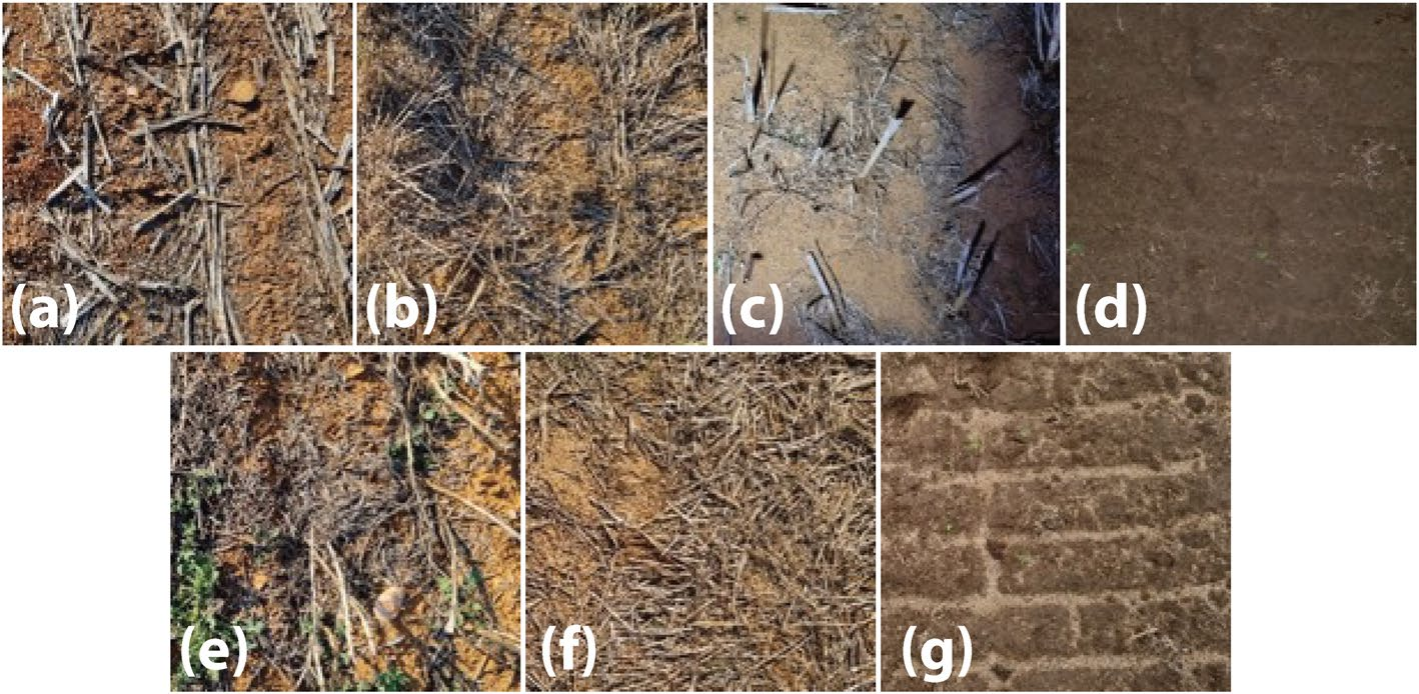
Representative images of the variable background and lighting conditions for seven image collection scenarios, (**a**) HEN1, (**b**) HEN2, (**c**) NIGHT1, (**d**) NIGHT2, (**e**) WAG1, (**f**) WAG2 and (**g**) COB1, used to evaluate the performance of colour-based weed detection.

Video data were collected in cropping fields with varying weed plant morphology and density, and different background conditions of soil colour, stubble and lighting for the validation of OWL using four separate colour-based algorithms. The collection of data was designed to replicate the in-field video environment of the OWL unit as closely as practicable. Landowners provided field access and permission to record videos in each field transect. Videos were collected using a handheld apparatus, which enabled concurrent collection of video data from two Raspberry Pi HQ cameras. Algorithms were selected via a rotary switch for each transect. Both computer-camera pairs were powered with a 12 V battery and a 5 V, 5 A voltage regulator (POLOLU-4091; Pololu Corporation). Recorded videos as well as average frame rates were stored on the onboard 64 GB micro-SD card and offloaded after each data collection. Real time clock modules (ADA3386; Adafruit Industries) ensured accurate timestamps of recorded videos. Five transects were recorded in daylight at five distinct field locations (Fig. 4), with a further two sites (including one used for daylight collection) used for collection of video data under artificial illumination with a Stedi C-4 Black Edition LED Light Cube. The 40 W light provides 4,200 Lm with a colour temperature of 5700 K and similar field of illumination to the FOV of the camera. The field sites represented likely use cases for fallow weed control, including canola, barley and wheat stubble and tilled soil at six locations in southern New South Wales, Australia (Table 2). Transects of 50 m were traversed by walking at a target speed of approximately 4 km h^−1^, with the walking time for each transect recorded to determine true average speed (Table 2). Based on the 1 m FOV of the camera each transect covered a total area of 50 m^2^, which was used to calculate weed density. The authors undertook in situ visual inspection of growth stage and identification of the weeds growing in the field at each of the sites where videos were collected. As all weeds identified are commonly occurring species with recognizable features no plant specimens were collected for subsequent formal identification (Table 2).

**Table 2:** Summary of field locations, weed species, background conditions, weed growth stage range and image collection speeds (n = 5) in fields used for video data collection and analysis.

Field ID	Location	Coordinates	Light conditions	Background	Weeds present	Weed density (plants m^−2^)	Weed growth stages	Average speed (m s^−1^ ± SE)
HEN1	Henty, NSW	− 35.517102, 147.034436	Clear, morning full sun	Canola stubble, red–orange soil	annual sowthistle (*Sonchus oleraceus*), volunteer canola (*Brassica napus*), annual ryegrass (*Lolium rigidum*), volunteer faba bean (*Vicia faba*)	3.1	2-leaf to flowering	1.14 ± 0.02
HEN2	Henty, NSW	− 35.517102, 147.034436	Clear, afternoon full sun	Heavy wheat stubble, red soil	Volunteer wheat (*Triticum aestivum*), annual sowthistle, annual ryegrass	9.3	2-leaf to late tillering	1.16 ± 0.01
WAG1	Wagga Wagga, NSW	− 35.056986, 147.351146	Clear, morning full sun	Lupin stubble, red–orange soil	Volunteer narrowleaf lupins (*Lupinus angustifolius*), annual sowthistle	18.7	2-leaf to flowering	1.14 ± 0.01
WAG2	Wagga Wagga, NSW	− 35.056986, 147.351146	Clear, morning full sun	Grazed barley stubble	Volunteer barley (*Hordeum vulgare*), annual sowthistle	3.3	2-leaf to 8-leaf	1.24 ± 0.03
COB1	Cobbitty, NSW	− 34.021914, 150.662655	Overcast	Dark brown soil, freshly tilled, no soil cover	Wild radish (*Raphanus raphanistrum*), fumitory (*Fumaria officinalis*), large crabgrass (*Digitaria sanguinalis*), billygoat weed (*Ageratum conyzoides*), stagger weed (*Stachys arvensis*)	9.8	Cotyledon to 6-leaf	1.07 ± 0.01
NIGHT1	Culcairn, NSW	− 35.667692, 147.036800	Night	Canola stubble	annual sowthistle, khaki weed (*Alternanthera pungens*), awnless barnyardgrass (*Echinocloa colona*), annual ryegrass, common catsear (*Hypochaeris radicata*)	9.6	2-leaf to flowering	1.23 ± 0.01
NIGT2	Cobbitty, NSW	− 34.021914, 150.662655	Night	Dark brown soil, freshly tilled, no soil cover	Wild radish, fumitory, large crabgrass, billygoat weed, stagger weed	7.8	Cotyledon to 6-leaf	0.83 ± 0.01

### In field validation and evaluation of weed detection algorithms

**Figure 5:**
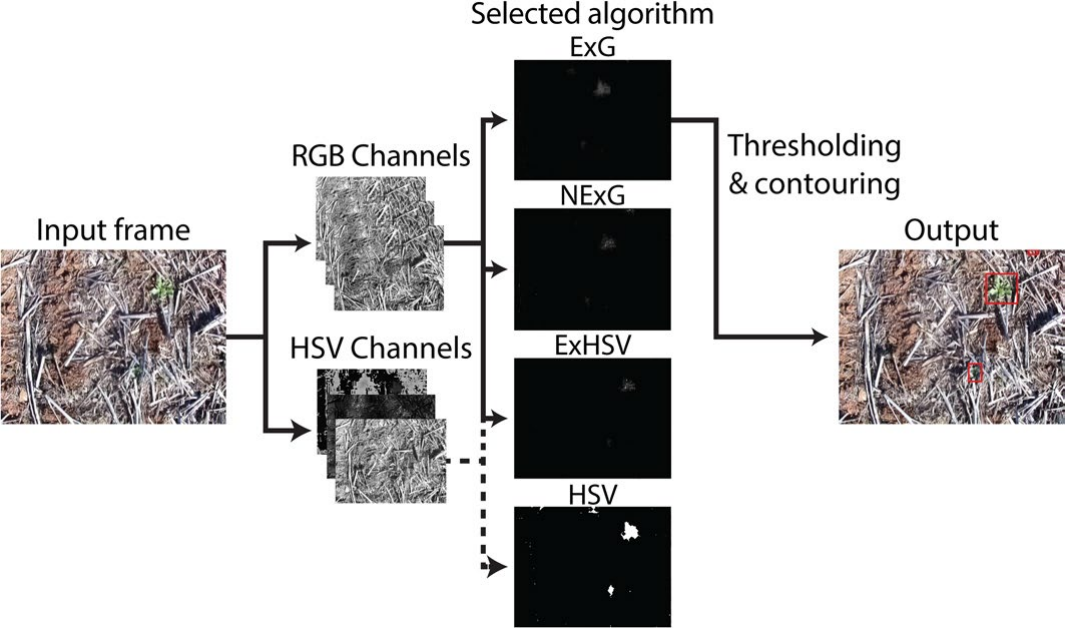
Overview of the frame-by-frame analysis process. Each 416 × 320 image is split into either red, green and blue (RGB) or hue, saturation and value (HSV) channels, and the ExG, NExG, ExHSV or HSV algorithm applied. A defined threshold is applied to the processed image followed by an adaptive threshold on the result (except HSV which is already binary) followed by contour detection and the generation of minimum enclosing rectangles for weed centre calculation.

Four colour-based algorithms that exploited the greenness of weeds for detection were used to validate OWL hardware. The algorithms were selected based on ease of implementation, alignment with human colour perception^56^ and use in weed detection and vegetation segmentation^19,66–68^: (1) raw excess green (ExG); (2) normalised excess green (NExG); (3) hue, saturation, value (HSV). A combined ExG and HSV (4) algorithm (ExHSV) (Fig. 5) was implemented to manage the sensitivity of ExG to expected changes in brightness in field conditions. Algorithm indices were calculated on a frame-by-frame basis by splitting each image into colour channels. Thresholds were then applied in conjunction with morphological operations to remove image noise. Remaining areas were marked as positive detections. All image processing software was written in Python 3.6^69^ and completed using OpenCV^70^, NumPy^71^ and imutils^72^ in addition to inbuilt libraries.

For (1) ExG, the algorithm is adapted from Woebbecke et al.^18^:

1 $$ExG= 2G-R-B$$

where G, R and B represent the raw pixel intensities for the green, red and blue channels, respectively of the digital camera image. The raw channel intensities may be influenced by environmental lighting conditions, which can be minimised by normalising individual channel values by the sum of all channels:

2 $$r=\frac{R}{R+G+B}, g= \frac{G}{R+G+B}, b= \frac{B}{R+G+B}$$

The resultant values from 0 to 1 were scaled by 255. The (2) NExG algorithm was calculated using the normalized channel intensities:

3 $$NExG=2g-r-b$$

On the resultant grayscale image within the threshold bounds of both ExG and NExG, an adaptive threshold was applied generating a binary (black and white only) masking image.

For (3) HSV, the colour space was converted from the RGB colour space to HSV using inbuilt OpenCV functions, with thresholds applied to each of the channels (Table 3). The minimum and maximum values for each threshold were manually selected to minimise over sensitivity, whilst maximising the number of true positives. The combined (4) ExHSV algorithm required a value to be both within the NExG threshold and the HSV binary region.

**Table 3:** Threshold parameters used for each of the four algorithms, where relevant.

Parameters	Day						Night					
	ExG/NExG		ExHSV		HSV		ExG/NExG		ExHSV		HSV	
	Min	Max	Min	Max	Min	Max	Min	Max	Min	Max	Min	Max
ExG	13	200	13	200	–	–	29	200	29	200	–	–
Hue	–	–	30	92	35	84	–	–	30	92	45	80
Saturation	–	–	4	250	10	220	–	–	10	250	75	200
Value	–	–	15	250	50	200	–	–	60	250	46	240
Object size (pixels)	10	–	10	–	10	–	10	–	10	–	10	–

Values represent pixel intensities for zero-indexed 8-bit arrays with a range of 0–255. Pixel values that did not sit within the ranges were excluded, hence leaving only green pixels as the detected object. Separate thresholds were used for the day and night videos. A minimum object size was implemented to reduce noise and is based on the area of each detected object. Values were selected manually to optimize algorithm performance.

For the resultant binary images, the within-image coordinates and size of each detection were returned for allocation to specific activation zones (Fig. 1a). The frame rates for each algorithm were recorded over five separate transects each 60 s in duration to ensure consistency in reporting.

### Video analysis of algorithm performance

Videos collected by the handheld video apparatus at a resolution of 416 × 320 pixels were analysed using standard, desktop computers on a frame-by-frame basis. Each frame was processed using the respective algorithms and threshold settings (Table 3), whereby detections were displayed as red boxes (Fig. 5). A separate, high-definition video of the transect was used to count all weeds within the field of view for the ground-truth data. True and false positives were recorded by comparison with the high-definition video. Algorithm performance was measured by calculating recall (Eq. 4) and precision (Eq. 5). Recall refers to the proportion of weeds detected when compared with all those present in the transect. Precision refers to the proportion of detections that were correct.

4 $$Recall= \frac{True\; Positives}{Total\; Weeds\; Present}$$

5 $$Precision= \frac{True\; Positives}{True\; Positives+False\; Positives}$$

### Statistical analysis

A one-way analysis of variance (ANOVA) was used to compare means of precision, recall and framerates across all fields implemented in RStudio^73,74^. The Shapiro–Wilkes test (P > 0.05) was used to test for normality. Precision data were transformed using a fifth power due to a negative skew in the distribution. Homogeneity of variance for recall and the transformed precision data were assessed with the Bartlett (P > 0.05) and Fligner–Killeen tests (P > 0.05). Data were visualised with ggplot2^75^ in RStudio. Pair-wise comparisons of framerates were made with the Agricolae package^76^. Illustrative figures were composed in Adobe Illustrator (v 24.4.1; Adobe Inc., San Jose, CA, USA).
